# Middlesex Hospital

**Published:** 1905-07-08

**Authors:** 


### MIDDLESEX HOSPITAL.

### THE KING'S FUND AND THE MEDICAL SCHOOLS.

A Special Court of Governors of the Middlesex Hospital was held on Friday, June 30th, to consider what steps should be taken to meet the resolution of King Edward's Hospital Fund for London with reference to lending support to the medical schools from funds belonging to the hospital. The Duke of Northumberland, president of the hospital, took the chair, and the meeting was well attended.

To each of the governors there was issued, with the notice convening the meeting, the following statement respecting the relationship between the hospital and its medical school, signed by Lord Cheylesmore, the chairman of the Weekly Board of Management.

The medical school of the hospital was established in pursuance of a resolution of a General Court of Governors held in the month of May 1835, and its existence, which has been continuous since that time, has been and is of great assistance to the hospital in the relief of its sick and indigent patients. It has also indirectly yielded great advantages to the public. Up to the year 1896 the school was managed by the medical staff of the hospital, who received the fees paid by the students, which were till then sufficient to meet the expenses of carrying on the school; but owing to the increased length and requirements of the curriculum it was then found necessary to procure the assistance of paid teachers to aid in the instruction given by the staff. There had also been a considerable falling off in the number of the students in all the Metropolitan schools, largely due to the competition of the provincial schools.

Under these circumstances the Weekly Board were informed by the Medical Staff that they could no longer carry on the school without financial assistance. The hospital authorities were convinced that the loss of the school would damage the hospital, and after prolonged conferences a scheme was agreed on by which the school was to be amalgamated with and carried on directly by the hospital. This scheme, having been submitted to a Court of Governors and approved of by them, was carried into effect, and the school has since 1896 been carried on by the hospital with the assistance of the staff and the necessary paid teachers.

The average yearly expense incurred by the hospital during the nine years which have since passed has only amounted to between three and four per cent. of the total ordinary annual expenditure of the hospital. During these nine years full accounts of all the expenditure of the hospital, which stated clearly the amounts spent on the school, have been placed before the general meetings of the governors at the end of each financial year, and these accounts have been approved of and adopted by them.

The Executive Committee of the King's Fund have passed the following resolution:—

"That the Executive Committee, having considered the recommendation of Sir Edward Fry's Committee (par. 27), advises the General Council to notify to the hospitals that no grants will be made from the King's Fund as from and after January 1st, 1906, to any hospitals which make payments to, or on account of, their medical schools out of general funds subscribed for the relief of the sick poor."

And it is probable that a similar resolution may shortly be passed by the Hospital Sunday Fund. The annual grants hitherto made by these two Funds to the hospital amount to a considerable sum, which the hospital can ill afford to lose, and the object of the special meeting of the governors which has been summoned is to adopt such steps as may enable the school to be carried on by the hospital without losing these grants.

The only alteration required for this purpose is that instead of the payments towards carrying on the school being approved and adopted as heretofore, after they have been made, the authority to make them should be given before they are made, and that is the authority which we are asking the friends of the hospital and those interested in its welfare to give.

Unless such authority is given by a sufficient number of subscribers it will be necessary either to discontinue the school or to lose the grants above referred to.

The abolition of the school, in addition to the loss of prestige which would accrue, would entail a large annual increase in the expenses of the hospital, as the work now done by the students in the wards would have to be discharged by paid officials.

The Chairman said the reason for the meeting was that the committee of King Edward's Fund had intimated to the hospitals of London that they did not intend to give grants after the present year to any hospital which contributed to the upkeep of its medical school out of the general funds, unless a distinction was made between contributions for the sick poor and for the medical schools. He did not wish, as a layman, to pronounce an opinion on what was undoubtedly a very difficult question, as between the medical schools and the hospitals. It was not possible to read the evidence given to the committee, and the correspondence on the subject, without perceiving that there was great room for legitimate difference of opinion, but it was clear that upon the connection between the medical schools and the hospitals, and the training which the medical schools could afford, turned not only the welfare of the sick in London, but of the sick and suffering throughout the length and breadth of the land, and that the whole medical profession would be affected by the decision come to. Perhaps he would not be considered guilty of presumption if he said there seemed to have been something of haste, something drastic, in the position assumed by King Edward's Fund in this matter. Be it right or be it wrong, he thought somewhat more tentative and less sudden action would have been better. But he deprecated any expression which might lead to complications; it was far better they should avoid all opportunities for friction as far as they could. They must remember it was not so much a question of the hospitals and medical schools as institutions in themselves, as of the sick and suffering, and the medical profession throughout the country. It was worth while that they should suppress any irritation they might feel, and bottle up some steam they would like to let off. He trusted they would be able to arrive at a solution so that all might work together for the common good. He called upon Sir Richard Powell to move the first resolution.

Sir Richard Douglas Powell said he wanted first to express his sense of appreciation of the loyalty to the school which had been shown by the Board of Management, and of the steadfast courage with which they had assumed a position which he was sure was of advantage to the public. That medical schools should be maintained seemed to him beyond question; they were a necessary part of a complete hospital. The object of a hospital was not only to do its immediate work but to further the science of medicine on which hospitals were founded. Without the school, the teaching, and the presence of such a learned audience as was thus provided he did not think the physicians and surgeons at the bedsides could be at their best. That a student-public was always present was unquestionably for the advantage of the hospitals and of the patients who came there. The rich public reaped the reflected benefit from hospital research and teaching, and the poor reaped the benefit through the greater and more thorough investigation of disease which occurred in hospitals where there was a medical school. The cost of the medical school to the Middlesex had been from 3 to 4 per cent. of its total expenditure since 1896, and he was quite sure that if it were taken away—if they could imagine the hospital going on without it—he would put 7 to 10 per cent. as the addition to the expenditure which would be thereby necessitated. It was of immense importance that the students should be kept in touch with the patients; and not only the students, but the public would suffer if the absurd idea gained acceptance that in two years they could acquire that insight and sympathy with patients which was included in diagnosis. He thought it would be an excellent thing if a strong committee of surgeons and physicians could be formed to look after the interest of the public in regard to the education and status of their future medical advisers. He moved:—

> That it is the desire of the Special Court of Governors that the Medical School of this Hospital, which has been in existence for the last 70 years, should be maintained, and that such assistance as may be required for that purpose should be provided by the Hospital or by a special fund raised for the purpose.

This was seconded by Mr. Henry Morris, senior surgeon of the hospital. He considered it impossible that the fees should be raised, for the bulk of medical students came from families of moderate means, and most of them had only moderate prospects. If the fees were raised it would be necessary for the Government or the ratepayers to subsidise the schools. London was the only place in the world where they were self-supporting; all medical schools on the Continent were State-supported, and in the provincial cities of England they were supported by grants or from endowments. Personally he would like to see a fund set aside to which money for the schools could be directly contributed.

The resolution was carried unanimously, as was also the following, proposed by Lord Derby and seconded by Lord Cheylesmore:—

> That in order to meet the requirements of King Edward's Hospital Fund the Weekly Board be authorised to set aside out of the annual subscriptions such sums as may from time to time be required to effect such maintenance, and that in evidence of such authority subscribers be asked and recommended to sign and return to the hospital the accompanying form.

Lord Cheylesmore mentioned that of 504 governors only five so far had declined to give the required sanction, and in reply to a question by the Hon. Arthur Stanley, M.P., he said he believed the alterations would meet the wishes of the King's Fund. He had been given to understand that if they got the consent of their governors that would be accepted.

A further resolution was adopted, sanctioning the expenditure of £1,044 on improvements in connection with the operating theatre, and the meeting terminated with a vote of thanks to the Duke of Northumberland for presiding.

